# Evaluation of Complex Drug Interactions Between Elexacaftor-Tezacaftor-Ivacaftor and Statins Using Physiologically Based Pharmacokinetic Modeling

**DOI:** 10.3390/pharmaceutics17030318

**Published:** 2025-03-01

**Authors:** Eunjin Hong, Peter S. Chung, Adupa P. Rao, Paul M. Beringer

**Affiliations:** 1 College of Pharmacy, CHA University, 335 Pangyo-ro, Bundang-gu, Seongnam-si 13488, Gyeonggi-do, Republic of Korea; 2 Gradute School of Clinical Pharmacy, CHA University, 335 Pangyo-ro, Bundang-gu, Seongnam-si 13488, Gyeonggi-do, Republic of Korea; 3 Division of Pulmonary and Critical Care Medicine, Department of Medicine, Keck School of Medicine, University of Southern California, 1975 Zonal Ave, Los Angeles, CA 90033, USA; 4 USC Anton Yelchin CF Clinic, 1510 San Pablo St., Los Angeles, CA 90033, USA; 5 Department of Clinical Pharmacy, Mann School of Pharmacy and Pharmaceutical Sciences, University of Southern California, 1985 Zonal Ave, Los Angeles, CA 90033, USA

**Keywords:** physiologically based pharmacokinetic (PBPK), drug–drug interactions, cystic fibrosis (CF), cystic fibrosis transmembrane conductance regulator (CFTR) modulator, Statin, cytochrome P450 isoenzymes (CYP), organic anion-transporting polypeptides (OATPs)

## Abstract

**Background/Objectives:** The increasing use of statins in people with cystic fibrosis (CF) necessitates the investigation of potential drug–drug interactions (DDI) of statins with cystic fibrosis transmembrane conductance regulator (CFTR) modulators, including elexacaftor, tezacaftor, and ivacaftor (ETI). The interactions may involve the potential inhibition of cytochrome P450 isoenzymes (CYPs), organic anion-transporting polypeptides (OATPs), and Breast Cancer Resistance Protein (BCRP) by ETI. This presents a therapeutic challenge in CF due to the potential for elevated statin levels, consequently heightening the risk of myopathy. This study aimed to predict potential DDIs between statins and ETI using a physiologically based pharmacokinetic (PBPK) modeling approach. **Methods:** We performed in vitro assays to measure the inhibitory potency of ETI against OATPs and CYP2C9 and incorporated these data into our PBPK models alongside published inhibitory parameters for BCRP and CYP3A4. **Results:** The PBPK simulation showed that atorvastatin had the highest predicted AUC ratio (3.27), followed by pravastatin (2.27), pitavastatin (2.24), and rosuvastatin (1.83). **Conclusions:** Based on these findings, rosuvastatin appears to exhibit a weak interaction with ETI, whereas other statins exhibited a moderate interaction, potentially requiring appropriate dose reductions. These data indicate potential clinically significant DDIs between ETI and certain statins, which warrants a clinical study to validate these findings.

## 1. Introduction

The utilization of statins is increasing in people with cystic fibrosis (CF). Their proven efficacy in mitigating chronic lung allograft dysfunction (CLAD), the most common and fatal complication of post-lung transplant, has been shown to reduce morbidity and mortality [1,2]. Beyond the primary role in reducing cholesterol levels, statins exhibit anti-inflammatory activities, thereby reducing chronic inflammation and subsequently impeding the progression of CLAD [2,3]. Moreover, an increased incidence of diabetes and obesity in people with CF may also warrant greater use of statins [4,5,6]. The introduction of Cystic Fibrosis Transmembrane Conductance Regulator (CFTR) modulators, specifically the triple combination of elexacaftor, tezacaftor, and ivacaftor (ETI, TRIKAFTA^®^), has led to an increased life expectancy in people with CF [7]. With the aging CF population, the prevalence of comorbidities, including obesity, high cholesterol, and cardiovascular issues, has significantly increased, consequently amplifying the use of statin therapy [6].

While statins are generally considered safe, they are susceptible to drug–drug interactions (DDIs) that augment their systemic exposure, thereby increasing the risk of statin-associated muscle symptoms [8]. These interactions involve a complex interplay between drug-metabolizing enzymes, primarily cytochrome P450 isoenzymes (CYP) 3A or 2C, and transporters, including organic anion-transporting polypeptides (OATPs) and Breast Cancer Resistance Protein (BCRP), which collectively influences the disposition of individual statins [9]. The pharmacokinetics of statins may be altered in people with CF receiving CFTR modulator therapy, given the potential OATP1B1/3 inhibitory activity of elexacaftor and BCRP inhibitory activity of elexacaftor and ivacaftor [10]. Moreover, all components of ETI potentially inhibit CYP2C9 and/or CYP3A4 [10], thereby introducing additional complexity to the estimation of interactions with statins. This presents a therapeutic challenge in people with CF due to the potential for elevated statin levels with concurrent administration of ETI, consequently heightening the risk of myopathy.

The National Lipid Association Scientific Statement on Statin Intolerance highlights statin DDIs as a key modifiable risk factor for preventing statin-associated muscle symptoms [11]. Consequently, the identification and avoidance of clinically significant statin DDIs are critical to ensure their safe use in the CF population. However, there is currently a lack of clinical data elucidating the interactions between ETI and statins, and no specific dosing guidelines have been established. Current prescribing information for ETI highlights the necessity for the cautious use of statins with appropriate monitoring due to the potential elevation of drug exposure, but detailed guidance remains lacking. Furthermore, seven statins have received approval from the FDA; however, their elimination profiles and specificity to certain CYP enzymes and OATP, as well as BCRP transporters, vary significantly. Hence, there is an urgent need to quantify the DDI magnitude for each statin when concomitantly used with ETI and to develop comprehensive guidance on dose adjustment of individual statins to minimize DDIs with ETI.

This study aimed to predict the pharmacokinetics of statins when concomitantly administered with ETI. Utilizing a physiologically based pharmacokinetic (PBPK) simulation-based approach, the transporters and enzyme-mediated drug interactions associated with statins and ETIs were evaluated. This modeling framework enabled the prediction of intricate mechanisms underlying DDIs involving OATP1B1, OATP1B3, BCRP, CYP3A4, and CYP2C9. Also, the study aimed to predict potential dose modifications of statins when co-administered with ETI, a provisional approach that could be employed pending the completion of clinical studies. The present study contributes to improving CF patient care by offering the tool to assess and potentially mitigate transporters and enzyme-mediated drug interactions involving CFTR modulators.

## 2. Materials and Methods

### 2.1. In Vitro Assay for Transporter and CYP Inhibition Potential

#### 2.1.1. In Vitro OATP1B1 and OATP1B3 Inhibition Potential of Elexacaftor

To simulate the transporter-mediated DDI, we investigated the inhibitory potential of elexacaftor against OATP1B/3 transporters with the probe substrate (estradiol-17β-glucuronide) and incorporated the obtained parameters into the previously developed elexacaftor model [12]. OATP1B1 or OATP1B3 over-expressing HEK293 cells were utilized to evaluate the inhibitory effect of elexacaftor for OATPs. The assay was performed by Pharmaron (Beijing, China). Briefly, the pre-incubation solution was prepared by diluting elexacaftor or rifampicin (positive control) in DMSO and then further diluting it with transport buffer. The final concentrations of elexacaftor were 0.3, 1, 3, 10, 30, and 100 μM, and the final concentrations of rifampin were 0.03, 0.1, 0.3, 1, 3, and 10 μM. The cells were incubated with the pre-incubation solution for 30 min at 37 °C, followed by the incubation solution containing estradiol-17β-glucuronide (a substrate of OATPs). The incubation solution was removed after a 2-min incubation, whereupon the cells were washed three times. The uptake activity of the transporter was determined by measuring the uptake of estradiol-17β-glucuronide into cells using UPLC-MS/MS. The IC_50_ was determined to be the concentration that produces half-maximal inhibition of the transport of estradiol-17β-glucuronide. The Ki values were calculated using the Cheng–Prusoff equation, Ki = IC_50_/(1 + [S]/Km), where [S] is the substrate concentration and Km is the Michaelis constant.

#### 2.1.2. In Vitro CYP2C9 Inhibition Potential of Ivacaftor

To simulate potential DDIs mediated by CYP2C9 inhibition, we investigated the inhibitory potential of ivacaftor against CYP2C9 and incorporated the obtained parameter into the previously developed ivacaftor model [13]. The inhibition of CYP2C9 was evaluated by Pharmaron (Beijing, China) using pooled human liver microsomes (HLM), following an established in vitro assay protocol [14]. Ivacaftor and ticrynafen (positive control) were diluted to final concentrations of 0, 0.001, 0.005, 0.01, 0.05, 0.25, 1, 10, and 50 μM. The compounds were incubated with diclofenac, a CYP2C9 substrate, and the assay was performed in triplicate. The conversion of diclofenac by CYP2C9 to the corresponding metabolite was detected by LC-MS/MS methods, and a decrease in the formation of the metabolite compared to the vehicle control was used to calculate an IC_50_. The curve was fitted using XLfit 5.5.0.5 (4 Parameter Logistic model, equation 201: parameter C equivalent to IC_50_). The Ki values were calculated using the Cheng–Prusoff equation, Ki = IC_50_/(1 + [S]/Km), where [S] is the substrate concentration and Km is the Michaelis constant.

#### 2.1.3. LC-MS/MS Assay of OATP1B and CYP2C9 Probe Substrates

To measure the estradiol-17β-glucuronide (OATP1B substrate), the system consisting of a Shimadzu LC-40D XS (Shimadzu Corporation, Kyoto, Japan) was utilized. The condition is as follows: mobile phase A 0.1% formic acid in water, mobile phase B 0.1% formic acid in acetonitrile; gradient, 30% B (0.05 min), 95% B (0.8–1.4 min), 30% B (1.5–2.0 min); flow rate, 0.6 mL/min; column temperature, 40 °C; and injection volume, 5 μL. The MS analysis was performed on a Sciex 6500 triple quadrupole mass spectrometer (AB Sciex LLC, Framingham, MA, USA) equipped with an ESI source. The detection was conducted using the MRM scan type in negative ion mode. The instrument-dependent parameters were ionspray voltage, −4.5 kV; ion source temperature, 550 °C; curtain gas, 30 L/min; and collision gas, 8 L/min.

The conversion of diclofenac by CYP2C9 to the corresponding metabolite was detected by the system consisting of a Shimadzu LC-40D XS (Shimadzu Corporation, Kyoto, Japan) and an API 4000/Triple Quad 4500 mass spectrometer (AB Inc., Richmond Hill, ON, Canada) equipped with an ESI source. The detection was conducted using the MRM scan type in positive ion mode. The instrument-dependent parameters were ionspray voltage, +5.5 kV; ion source temperature, 500 °C; curtain gas, 30 L/min; and collision gas, 6 L/min.

### 2.2. PBPK Model Development

#### 2.2.1. PBPK Models of ETI

The PBPK models were implemented using the Simcyp Simulator (version 22; Certara, Sheffield, UK). To simulate the complex inhibitory effects of ETI on OATP1B1/3, BCRP, and various CYP enzymes, we incorporated in vitro-derived inhibition parameters into the previously published model of ETI [12,13]. The original ETI model included an elexacaftor Ki of 5.45 μM against CYP2C9, tezacaftor Kis of 12.5 μM and 13.5 μM against CYP3A4 and CYP2C9, respectively, and an ivacaftor Ki of 11.56 μM against CYP3A4. In the current study, we supplemented these parameters with additional inhibitory potency data: OATP1B1/3 inhibition by elexacaftor and CYP2C9 inhibition by ivacaftor, both derived from in vitro assays. We also incorporated the published IC_50_ of 7.13 μM for BCRP inhibition by ivacaftor [15]. Using the Cheng–Prusoff equation, with a substrate concentration of 1 μM and Km of 3.2 μM, the Ki value against BCRP was calculated to be 5.43 μM. A detailed summary of the input parameters for elexacaftor, tezacaftor, and ivacaftor is provided in Supplementary Tables S1–S3.

#### 2.2.2. PBPK Models of Statins

**Figure 1:**
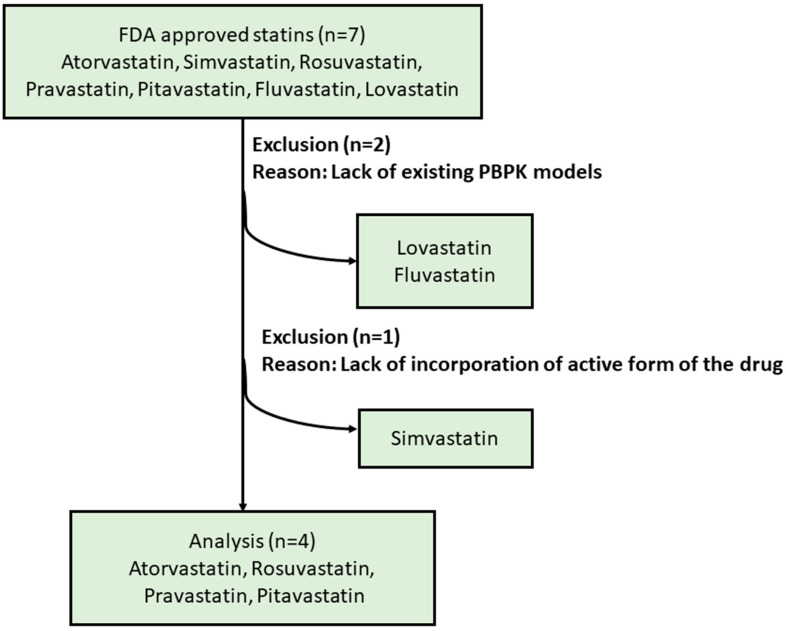
Flowchart illustrating the inclusion of four statins employed for DDI studies.

Rosuvastatin, pravastatin, atorvastatin, and pitavastatin were used for DDI simulations with ETI. The vendor-verified statin compound files in Simcyp^®^ (version 22) library were used. Lovastatin, fluvastatin, and simvastatin were excluded due to the lack of availability of PBPK models for these compounds, either in the Simcyp compound library or the published literature (Figure 1). In the case of simvastatin, while a PBPK model is available, it does not include the active form of the prodrug simvastatin (simvastatin acid).

#### 2.2.3. Population Model

In the default healthy population library file (Sim-Healthy volunteers) provided in Simcyp^®^, the distribution of ages and proportion of females were adjusted to reflect the demographics of the CF population based on the patient registry 2020 annual report published by the Cystic Fibrosis Foundation [16]. Specifically, the frequency in the population aged 18–21 years was adjusted from 4.5% in the healthy population to 13.1% in CF. Also, the proportion of females was adjusted from 0.32 in the healthy population to 0.48 in CF. All other system parameters were kept as default, based on data demonstrating that the PK parameters of ETI do not differ between healthy adults and people with CF [17,18,19]. This population library file was used for all simulations. For trial design, we used a population size of 100 (10 trials and 10 subjects in each trial), aligning with commonly used sample sizes in PBPK modeling.

### 2.3. PBPK Model Verification

#### 2.3.1. Pharmacokinetics Simulations

The plasma PK profiles of ETI following multiple dose administration of clinically relevant doses (elexacaftor 200 mg once daily, tezacaftor 100mg once daily, and ivacaftor 150 mg twice daily) were simulated to verify the performance of the PBPK models after incorporating several drug inhibition potentials. ETI was orally administered under fed conditions to mimic the clinical setting, where a fat-containing food is required for optimal absorption of ETI. The simulated data were compared to the observed plasma PK data in an adult CF population [19].

The accuracy of the area under the curve (AUC) and maximum plasma concentration (C_max_) prediction values were calculated as a ratio of mean observed over mean predicted values. Successful model performance was defined a priori by ratios of AUC and *C*_max_ within a two-fold range, as previously described [20,21].

#### 2.3.2. DDI Simulations with CYP3A4/2C9 Probes

Upon accurate recapitulation of the pharmacokinetics, the ivacaftor model was further evaluated using the clinical DDI data to confirm its suitability for assessing the CYP3A4 and CYP2C9 inhibition-derived DDIs. The OATP and BCRP transporter inhibition was not verified against clinical DDIs due to a lack of clinical studies.

We compared the simulated DDIs with those observed in clinical studies with midazolam (substrate of CYP3A4) and ethinylestradiol (substrate of CYP3A4 and 2C9) [17]. The input parameters of these drugs are available in the compound library of Simcyp version 22: SV-Ethinylestradiol and Sim-Midazolam. For verification simulations, the dose and schedule of drugs were matched to the design of the corresponding clinical DDI study. For ivacaftor-midazolam, ivacaftor 150 mg was administered every 12 h for 6 days, and a single oral dose of midazolam 2 mg was administered on day 6. For ivacaftor-ethinylestradiol, ivacaftor 150 mg was administered every 12 h for 28 days, and ethinylestradiol 0.035 mg was administered once daily for 21 days. To quantify the DDIs, the geometric mean ratios of AUC or C_max_ in the presence or absence of ivacaftor were determined. The assessment of DDI prediction accuracy was based on whether the predictions fell within the bioequivalence range (0.80–1.25) of the observed data.

### 2.4. PBPK Simulations of Drug–Drug Interactions of Individual Statins with ETI

We conducted simulations to assess the DDIs of atorvastatin, pravastatin, rosuvastatin, and pitavastatin when concomitantly used with standard dose (elexacaftor 200 mg daily, tezacaftor 100 mg daily, and ivacaftor 150mg twice daily) ETI at a steady state. For the statins, simulations were performed using the second lowest recommended dose (atorvastatin 20 mg, pravastatin 20 mg, rosuvastatin 10 mg, and pitavastatin 2 mg daily). To ensure steady-state conditions, statins and ETI were co-administered for 20 days.

The DDI magnitude was categorized according to FDA guidance: an AUC ratio of statin less than 2-fold indicates a weak interaction, an AUC ratio between 2.0 to 5.0 indicates a moderate interaction, and an AUC ratio over 5.0 indicates a strong interaction. Based on an analysis conducted by the University of Washington Drug Interaction Solutions group, weak interactions (AUC ratio less than 2-fold) may not require dose adjustments, as suggested by drug labeling data [22].

## 3. Results

### 3.1. In Vitro Assay for OATP Transporters and CYP2C9 Inhibition Potential

#### 3.1.1. In Vitro Testing of Elexacaftor OATP1B1/3 Inhibition

**Figure 2:**
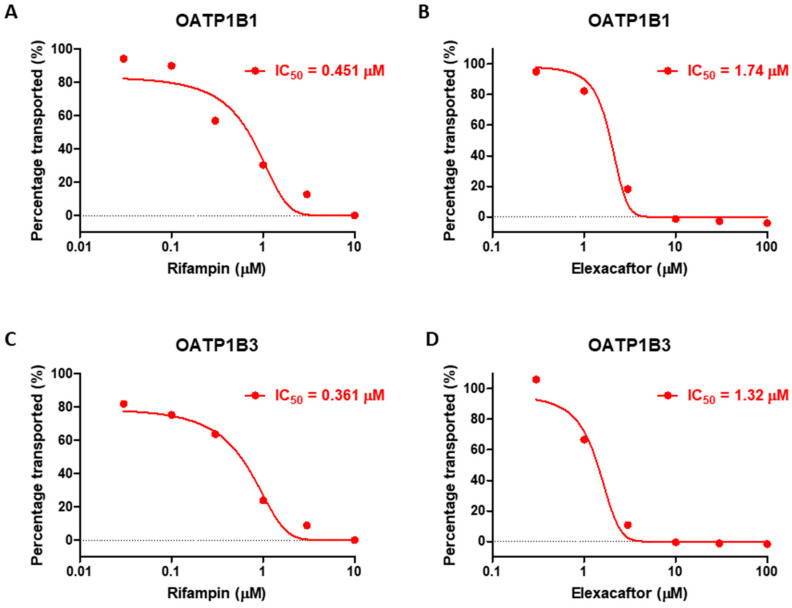
In vitro OATP1B1 inhibition by rifampin (**A**) and elexacaftor (**B**) and OATP1B3 inhibition by rifampin (**C**) and elexacaftor (**D**).

The IC_50_ of elexacaftor on OATP1B1 and OATP1B3 when estradiol-17β-glucuronide was used as in vitro probe substrate were 1.74 μM (90% CI: 1.56 to 1.93) and 1.32 μM (90% CI: 1.13 to 1.55), respectively (Figure 2). The [S] and Km were 5 μM and 6.8 μM for OATP1B1 and 10 μM and 13.1 μM for OATP1B3, respectively. The calculated elexacaftor K_i_ was 1.00 μM and 0.75 μM for OATP1B1 and OATP1B3, respectively.

#### 3.1.2. In Vitro Testing of Ivacaftor CYP2C9 Inhibition

**Figure 3:**
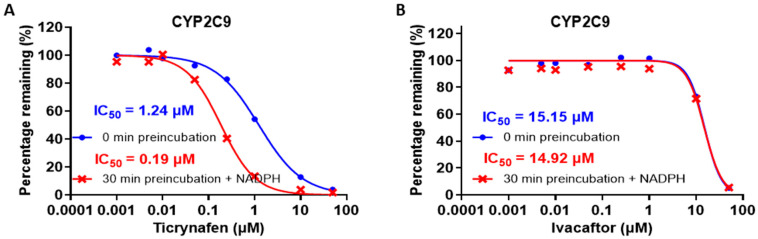
In vitro CYP2C9 inhibition by ticrynafen (**A**) and ivacaftor (**B**).

For the ivacaftor CYP2C9 inhibition assay, the absence of a shift in IC_50_ following a 30-min pre-incubation with NADPH suggests that the inhibition is NADPH-independent, whereas ticrynafen (positive control) showed NADPH-dependent inhibition (Figure 3). The IC_50_ values of ivacaftor obtained without preincubation and with preincubation were recorded as 15.15 μM and 14.92 μM, respectively. The [S] and Km values were 6 μM and 6.4 μM, respectively, and the calculated mean K_i_ was determined to be 7.75 μM.

### 3.2. PBPK Model Verifications

#### 3.2.1. PBPK Models of ETI Recapitulate Clinically Observed PK Profiles

Since several interacting parameters of ETI, including K_i_ against BCRP, OATP1B1/3, and CYP2C9 inhibition, were incorporated, the model predictive performance was re-assessed using observed plasma pharmacokinetic data from clinical trials [19]. The observed and simulated steady-state PK of ETI following a standard dose administration are summarized in Table 1. The predicted steady-state AUC and Cmax of ETI were in the range of 0.98–1.27 of the observed values, demonstrating the excellent performance of the model.

#### 3.2.2. PBPK Model of Ivacaftor Recapitulated Observed DDIs

To verify the CYP3A4 and CYP2C9 inhibition potential defined in the ivacaftor model, clinical DDI studies were simulated with CYP3A4 and/or CYP2C9 substrate drugs. The ivacaftor model recapitulated the observed DDIs with midazolam or ethinylestradiol, with the predicted AUC and Cmax ratio within the range of 0.93 to 1.11 of the observed values (Table 2).

### 3.3. DDI Simulation of ETI with Individual Statins

The PBPK models of ETI incorporating the in vitro inhibition of CYP2C9/3A4, BCRP, and OATP1B1/B3 were used to simulate the effect of ETI on the pharmacokinetics of statins. We calculated the *C*_max_ and AUC ratio of each statin in the presence and absence of ETI at steady-state (Table 3). The simulated geometric mean AUC ratio was highest for atorvastatin (3.27), followed by pravastatin (2.27), pitavastatin (2.24), and rosuvastatin (1.83). The simulated geometric mean *C*_max_ ratio was highest for atorvastatin (2.84), followed by pravastatin (2.01), pitavastatin (1.83), and rosuvastatin (1.84). Atorvastatin, pravastatin, and pitavastatin exhibited moderate interactions, with an AUC ratio exceeding 2.0, suggesting that dose reductions may be needed. Rosuvastatin exhibited a weak interaction, indicating that dose adjustment may not be required.

### 3.4. Potential Altered Dose of Statin When Concomitantly Used with ETI

**Figure 4:**
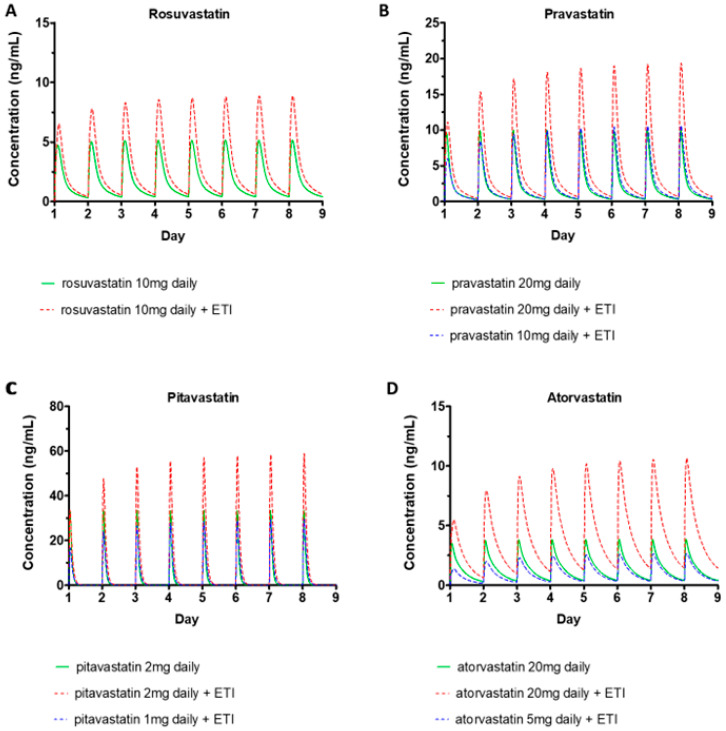
Plasma Concentration Profile of rosuvastatin (**A**), pravastatin (**B**), pitavastatin (**C**), and atorvastatin (**D**). Green: standard dose, Red: standard dose with ETI, Blue: reduced dose with ETI.

We next utilized the models to explore potential dose adjustments of statins when co-administered with ETI to mitigate the potential rise in drug concentrations induced by ETI. The dosing regimen was selected to target the AUC of the statins within the bioequivalence limit (0.80 to 1.25) relative to the statin administered alone. Simulation results suggested that a 50% dose reduction for pitavastatin or pravastatin was predicted to achieve AUC values between 113.1% and 123% of the statin administered alone (Figure 4). For atorvastatin, which showed the highest level of DDI among the statins examined, a 75% dose reduction (from 20mg daily to 5mg daily) was required to achieve an AUC equivalent to 82.6% of atorvastatin 20mg alone (Figure 4).

## 4. Discussion

The findings of this study shed light on the complex DDIs between statins and the CFTR modulators. The increasing use of statins in the CF population underscores the importance of investigating potential interactions between statins and ETI that may impact the safety and efficacy of the treatment in people with CF. The PBPK simulation approach identified significant differences in statin exposures with concomitant ETI, highlighting the need for further exploration in clinical trials.

The measured inhibition constants (K_i_) for elexacaftor on OATP1B1 and OATP1B3 using the probe substrate (estradiol-17β-glucuronide) were 1.00 μM and 0.75 μM, respectively, indicating that its potentials to impact the hepatic uptake of substrates mediated by these transporters are comparable. In contrast, the K_i_ values for gemfibrozil, serving as the probe inhibitor of OATP transporters, were 2.52 μM for OATP1B1 and 10.0 μM for OATP1B3, suggesting a relatively selective inhibition on OATP1B1. This disparity in OATP inhibition implies a differential effect on the uptake of statins, contingent upon their specificity for OATP1B1 and OATP1B3. The relative contributions of OATP1B1 and OATP1B3 to hepatic statin uptake are approximately 70% and 20% for rosuvastatin, 90% and 10% for pitavastatin, and more than 95% via OATP1B1 for both atorvastatin and pravastatin [23,24].

According to the clinical DDI data with gemfibrozil, the observed increase in AUC was noted as the lowest for atorvastatin (1.2-fold), followed by pitavastatin (1.5-fold), rosuvastatin (1.9-fold), and pravastatin (2.0-fold). This pattern differs from the predictions derived from our PBPK simulations with ETI, where rosuvastatin exhibited the least increase in AUC (1.8-fold), followed by pitavastatin (2.2-fold), pravastatin (2.3-fold), and atorvastatin (3.3-fold). This difference can be attributed to a dual rationale: firstly, the differing involvement of CYP enzymes in the metabolic pathways of each statin. Notably, atorvastatin, extensively metabolized by CYP3A4, demonstrated the most substantial increase in AUC when co-administered with ETI, despite exhibiting the lowest DDI with gemfibrozil. Secondly, the distinct relative participation of hepatic uptake facilitated by OATP1B1 and OATP1B3 may alter the disposition of statins by elexacaftor vs gemfibrozil. These preclude a straightforward reliance on previously observed clinical DDI data to determine the DDI between statin and ETI.

The primary limitation of the study is that the obtained in vitro OATP1B1 and OATP1B3 inhibition parameters for elexacaftor and BCRP inhibition parameters for ivacaftor were not verified against clinical DDIs due to the lack of clinical data. Additionally, the CYP2C9 inhibition incorporated into the ivacaftor model has been assessed with a single drug (ethinylestradiol) that is not classified as a sensitive index substrate. Owing to the lack of clinical studies, the current prescribing information suggests close monitoring when concomitantly administering the OATP and BCRP substrates (e.g., statins) or CYP2C9 substrates (e.g., warfarin) with ETI. In the absence of such data, our approach relied on the implementation of PBPK modeling to provide insights into statin interactions in people with CF receiving ETI, thereby bridging the gap with the need for the corresponding dosing guidelines. Further clinical studies are warranted to confirm the simulation results. Additionally, individual genetic polymorphisms, such as CYP2C9 or OATP1B polymorphisms, could influence the PK of specific statins and impact the extent of DDIs with ETI.

Another limitation of this study is that the simulation was based on a healthy population model due to the absence of population system parameters of CF. However, we incorporated changes in demographics that reflect the CF population, leading to changes in physiological parameters related to these covariates (e.g., liver weight). Previous studies indicate that differences in the pharmacokinetics of drugs in CF are attributed to variations in body composition and plasma protein concentrations resulting from nutritional deficiencies [25]. However, with recent improvements in CF care with the introduction of CFTR modulator therapy, the BMI of people with CF has increased over the years [16] to the extent it is now similar to that of healthy volunteers [26]. Indeed, the PK of ETI has been reported to be similar in both healthy volunteers and CF patients in recent studies [19]. Gastrointestinal processes are also known to be altered in CF, including increased gastric acidity and exocrine pancreatic insufficiency. However, there is no mechanism that fully explains the potential differences in the absorption of some drugs but not others [27]. A systematic review summarizing drug pharmacokinetics over a 20-year period concluded that there is currently insufficient evidence demonstrating that specific pathophysiological changes in CF predict altered drug absorption in people with CF [28]. Based on these findings, CF-related gastrointestinal changes were not incorporated into the model.

In conclusion, employing a PBPK modeling approach, we explored the DDI potential of various statins when used with ETI. Rosuvastatin appears to exhibit a weak interaction with ETI, whereas other statins exhibited a moderate interaction, potentially requiring appropriate dose reductions. The outcome of this study provides guidance on the potential dosing of statin therapy in people with CF while continuing to receive highly active CFTR modulators. While clinical validation is requisite, these findings serve as a valuable foundation for guiding the selection of statins for CF. In addition, this work provides valuable tools to address novel drug interactions mediated by OATP1B1/3, BCRP, and CYP2C9/3A4 inhibition by ETI, which could contribute to initial dose adjustment considerations while awaiting comprehensive clinical trial data.

## Figures and Tables

**Table 1:** Comparison of PK parameters between simulated and observed data for model verification of ETI.

PK Study		Steady-State PK Parameters				
Drug	Regimen		Simulated		Observed *	
			Cmax (mg/L)	AUC ^†^ (mg∙h/L)	Cmax (mg/L)	AUC ^†^ (mg∙h/L)
**elexacaftor**	**200 mg qd**	Mean	8.6	168.2	8.8	167.0
		CV(%)	45.9	48.9	24.6	30.2
		Simulated/ observed	0.98	1.01		
**tezacaftor**	**100 mg qd**	Mean	8.5	113.1	6.7	92.4
		CV(%)	44.1	48.7	20.8	25.8
		Simulated/ observed	1.27	1.22		
**ivacaftor**	**150 mg q12h**	Mean	1.5	12.6	1.3	12.1
		CV(%)	48.0	53.6	27.8	34.5
		Simulated/ observed	1.15	1.04		

* The observed data were obtained from [19]. ^†^ AUC (0–24 h) for elexacaftor and tezacaftor and AUC(0–12 h) for ivacaftor.

**Table 2:** Summary of the simulated vs. observed Geometric Mean Ratio (GMR) of PK parameters for co-administered drugs in the presence of ivacaftor.

Drug	PK Parameters	Simulated GMR (90% CI)	Observed * GMR (90% CI)	Ratio (Simulated/Observed)
Midazolam +/− ivacaftor	Cmax Ratio	1.53 (1.49, 1.56)	1.38 (1.26, 1.52)	1.11
	AUC Ratio	1.69 (1.64, 1.73)	1.54 (1.39, 1.69)	1.10
Ethinylestradiol +/− ivacaftor	Cmax Ratio	1.14 (1.13, 1.15)	1.22 (1.10, 1.36)	0.93
	AUC Ratio	1.17 (1.17, 1.18)	1.07 (1.00, 1.14)	1.09

* The observed GMR data were obtained from [17].

**Table 3:** Summary of the simulated Geometric Mean Ratio (GMR) of PK parameters of statins in the presence and absence of ETI.

DDI Study	PK Parameters	Simulated GMR (90% CI)
Atorvastatin +/− ETI	Cmax Ratio	2.84 (2.70, 2.99)
	AUC Ratio	3.27 (3.10, 3.44)
Pravastatin +/− ETI	Cmax Ratio	2.01 (1.93, 2.10)
	AUC Ratio	2.27 (2.18, 2.37)
Pitavastatin +/− ETI	Cmax Ratio	1.83 (1.76, 1.90)
	AUC Ratio	2.24 (2.14, 2.35)
Rosuvastatin +/− ETI	Cmax Ratio	1.84 (1.77, 1.91)
	AUC Ratio	1.83 (1.76, 1.90)

## Data Availability

Data is contained within the article or Supplementary Materials.

## Supplementary Materials

The following supporting information can be downloaded at https://www.mdpi.com/article/10.3390/pharmaceutics17030318/s1: Table S1: Parameters Used to Develop the Ivacaftor Model in Simcyp^®^ version 22 (Certara); Table S2: Parameters Used to Develop the Tezacaftor Model in Simcyp^®^ version 22 (Certara); Table S3: Parameters Used to Develop the Elexacaftor Model in Simcyp^®^ version 22 (Certara).
