# Supplementary material for: Evaluation of Complex Drug Interactions Between Elexacaftor-Tezacaftor-Ivacaftor and Statins Using Physiologically Based Pharmacokinetic Modeling
Source: Pharmaceutics. 2025 Mar 1;17(3):318. doi: 10.3390/pharmaceutics17030318 (PMC11945117; doi:10.3390/pharmaceutics17030318)
Supplement: Supplementary file 1 [file pharmaceutics-17-00318-s001.zip › pharmaceutics-3489115-supplementary.pdf]

Table S1. Parameters Used to Develop the Ivacaftor Model in Simcyp® version 22 (Certara)

**Parameter**	**Value**
Physiochemical properties	
Molecular weight (g/mol)	392.49
Log $P_{o:w}$	5.68
Compound type	Diprotic acid
pK_a 1	9.4
pK_a 2	11.6
B/P	0.55
fu_p	0.001
Absorption	
Absorption model	ADAM
Caco-2 permeability (10^{-06} cm/sec)	119
fu_{gut}	0.279
k_a (h^{-1})	5.188
F_g	0.5
$P_{eff,man}$ ($\times 10^{-4}$ cm/s)	11.9
Distribution	
Distribution model	Minimal PBPK model
Q (l/h)	3.752
V_{sac} (l/kg)	6.70×10^{-01}
V_{ss} (l/kg)	1.89
Elimination	
CL_{po} (l/h)	19.0
rCYP3A4 CL_{int} (µL/min/pmol of isoform)	19.9
Additional HLM clearance (µL/min/mg protein)	78.8
Interaction	
CYP3A4 K_i (µM)	11.56
fu_{mic}	0.001
CYP2C9 K_i (µM)	7.75
fu_{mic}	0.008
BCRP K_i (µM)	5.43
fu_{mic}	1

ADAM, advanced dissolution, absorption, and metabolism; B/P, blood-to-plasma ratio; CL_{int}, intrinsic clearance; CL_{po}, in vivo oral clearance; F_g, fraction escaping gut-wall elimination; fu_{gut}, fraction unbound in the enterocyte; fu_{mic}, fraction unbound in the in vitro microsomal incubation; fu_p, fraction unbound in plasma; HLM, human liver microsome; k_a, absorption rate constant; K_i, concentration of inhibitor that supports half maximal inhibition; Log $P_{o:w}$, logarithmic partition coefficient octonal:water; $P_{eff,man}$, effective permeability in man; pK_a, logarithm of acid dissociation constant; Q, inter-compartment clearance; V_{sac}, single adjusted compartment volume; V_{ss}, volume of distribution at steady state.

Table S2. Parameters Used to Develop the Tezacaftor Model in Simcyp® version 22 (Certara)

**Parameter**	**Tezacaftor**
Physiochemical properties	
Molecular weight (g/mol)	520.5
Log $P_{o:w}$	3.6
Compound type	Neutral
B/P	0.658
fu_p	0.009
Absorption	
Absorption model	First order
fu_{gut}	1
f_a	0.82
F_g	0.95
PCaco-2(10^{-6} cm/s)	4.70
Reference Compound	Multiple
Scalar	2.997
Distribution	
Distribution model	Minimal PBPK model
Q (l/h)	0.873
V_{sac} (l/kg)	0.113
V_{ss} (l/kg)	0.332
Elimination	
CL_{po} (l/h)	1.39
rCYP3A4 CL_{int} (µL/min/pmol of isoform)	0.175
Additional HLM clearance (µL/min/mg protein)	7.359
Interaction	
CYP3A4 K_i (µM)	12.5
fu_{mic}	0.876
CYP2C9 K_i (µM)	13.5
fu_{mic}	0.876
CYP2C19 K_i (µM)	22.5
fu_{mic}	0.876
CYP2C8 K_i (µM)	13
fu_{mic}	0.738

B/P, blood-to-plasma ratio; CL_{int}, intrinsic clearance; CL_{po}, in vivo oral clearance; f_a, fraction absorbed; F_g, fraction escaping gut-wall elimination; fu_{gut}, fraction unbound in the enterocyte; fu_{mic}, fraction unbound in the in vitro microsomal incubation; fu_p, fraction unbound in plasma; HLM, human liver microsome; K_i, concentration of inhibitor that supports half maximal inhibition; Log $P_{o:w}$, logarithmic partition coefficient octonal:water; Q, inter-compartment clearance; V_{sac}, single adjusted compartment volume; V_{ss}, volume of distribution at steady state.

Table S3. Parameters Used to Develop the Elexacaftor Model in Simcyp® version 22 (Certara)

**Parameter**	**Value**
Physiochemical properties	
Molecular weight (g/mol)	597.66
Log $P_{o:w}$	6.00
Compound type	Monoprotic acid
pK_a	5.04
B/P	0.55
fu_p	0.00704
Absorption	
Absorption model	First order
fu_{gut}	7.75e-4
f_a	0.82
F_g	1
k_a (h^{-1})	0.59
T_{lag} (h)	2.17
PCaco-2(10^{-6} cm/s)	3.08
Distribution	
Distribution model	Minimal PBPK model
Q (l/h)	10.05
V_{sac} (l/kg)	0.27
V_{ss} (l/kg)	0.62
Elimination	
CL_{iv} (l/h)	1.25
rCYP3A4 CL_{int} (µL/min/pmol of isoform)	0.233
Biliary CL_{int} (Hep) (µL/min/10^6)	4.56
Interaction	
CYP2C8 K_i (µM)	8.35
fu_{mic}	0.399
CYP2C9 K_i (µM)	5.45
fu_{mic}	0.399
OATP1B1 K_i (µM)	1.00
fu_{inc}	0.03
OATP1B3 K_i (µM)	0.75
fu_{inc}	0.03

B/P, blood-to-plasma ratio; CL_{int}, intrinsic clearance; CL_{iv}, in vivo intravenous clearance; f_a, fraction absorbed; F_g, fraction escaping gut-wall elimination; fu_{gut}, fraction unbound in the enterocyte; fu_{inc}, fraction unbound in the in vitro incubation; fu_{mic}, fraction unbound in the in vitro microsomal incubation; fu_p, fraction unbound in plasma; k_a, absorption rate constant; K_i, concentration of inhibitor that supports half maximal inhibition; Log $P_{o:w}$, logarithmic partition coefficient octonal:water; pK_a, logarithm of acid dissociation constant; Q, inter-compartment clearance; T_{lag}, lag time; V_{sac}, single adjusted compartment volume; V_{ss}, volume of distribution at steady state.
